# Book Review: Pratiquer l'ACT par le Clown [ACT through Clown]

**DOI:** 10.3389/fpsyg.2016.00296

**Published:** 2016-03-02

**Authors:** Mathieu Hainselin

**Affiliations:** CRPCPO, EA 7273, Université de Picardie Jules VerneAmiens, France

**Keywords:** acceptance and commitment therapy, cognitive behavior therapy, psychotherapy, hexaflex, happiness

“Let it go…Let it go” is one of the key message of the Acceptance and Commitment Therapy (ACT), as well as the Frozen movie. My 2 years old daughter Elise is very good at both: singing and let it go. She is also a great clown, such as another Elise Ouvrier-Buffet, a professional clown and co-author with Jean-Christophe Seznec of the book reviewed here (Seznec and Ouvrier-Buffet, 2014). Putting ACT and clown together is unusual and can be surprising…except if you are already familiar with both ACT and clown. Contact with the present moment, committed action, acceptance, self as context, values and defusion are some core characteristics of the clown, and the six psychological processes ACT seeks to strengthen (Hayes et al., 2006; Hacker et al., 2016). The clown is listening to his body, sensations and emotions, instead of focusing on distracting parasite thoughts. Therefore he is a great example for patients, as well as for therapists. The first author wrote his clown skills enhanced his psychiatrist job. Now he wants to play, laugh and have fun during sessions. If life includes pain and death, clown and therapist have to deal with it and, likewise, help other people to do so. Because of these similarities, ACT and clown can learn from each other; this was obviously the authors' guidelines for this eight chapters book.

Chapter 1 introduces us to the clown, using open questions. We learn the history of the white clown and Auguste, the role of fun and playing, his relationships with laugh (no need to be funny) and most importantly, other human being. Psychologists already familiar with ACT will enjoy the end of its chapter, dealing with clown values, very helpful to develop ACT skills.

Chapters 2 and 3 are respectively dedicated to ACT presentation and its models. Chapter 2 is a good reminder (or discover) of ACT and its roots: from meditation to Cognitive and Behavioral Therapies (CBT). To help the reader through the book, the authors quote famous or original quotes, including the Forrest Gump's chocolate box one to exemplify ACT principles. Hexaflex, matrix, C-ABC model and four squale tool are described in Chapter 3.

Chapter 4 is the ACT toolbox presentation, starting with metaphors. Specific tools for observation (including mindfulness), action, defusion (apples sorting), and emotions regulation. At this point, we can fully understand how ACT and clown overlap each other with psychological flexibility.

Chapter 5 starts with the classical ACT ocean metaphor and the surf spirit to set us up before exercises. Beyond classic ACT values, the focus is on playing, having fun and let our clown go. We also learn to say “yes” to anything, just like the Auguste. This is a very powerful skill also developed in improvization theater with the famous “yes, and…” (Bermant, 2013; Bernstein, 2014).

Chapter 6 is dedicated to practice. The first part contains illustrations for warm up and body expression exercises. We also learn clown skills to explore space, focus, voice, facial expressions, unlocking imagination, working with others and psychotherapeutic exercises from clown practice, as the “ask and no answer” game. In this exercise, people have to ask eccentric questions, so the answer can be no.

The two last chapters are the perspective sections. Chapter 7 introduces us to ACT and clown practice in different contexts, mostly at work, nonprofit organization and in hospital. Hospital clown associations grew up in the last 30 years on both side of the Atlantic: Big apple clown care in the USA or *Le Rire Médecin* in France. A French clown says how much her clown, being a third actor, changes the relationship between child in hospital and his parents. The clown makes parents laugh and the children enjoying the clown and their parents laughing. While clinical teams often want to comply with their supposed serious role, the clown helps them to improve their flexibility. While more studies need to confirm, clowns seem to have an impact in hospital, especially in pediatric practice (Fernandes and Arriaga, 2010; Finlay et al., 2014).

Chapter 8 is more personal. Authors let their thoughts go and explain how and why they wrote this book after a professional and personal journey. They became clown or psychiatrist because they love freedom, goodwill and humanism.

Overall, this book can be used as a toolkit for psychologists and psychiatrists, already familiar with ACT (or not), for their everyday practice. Anyone without particular psychological knowledge could benefit from this book, thanks to its exercises and clinical illustrations. The main concern about this book is that it is, for now, only available in French. I hope non-French speaking people reading this article will translate it to spread the ideas. Just say “yes.”

## Author contributions

The author confirms being the sole contributor of this work and approved it for publication.

### Conflict of interest statement

The author declares that the research was conducted in the absence of any commercial or financial relationships that could be construed as a potential conflict of interest.
